# A smart glove with integrated triboelectric nanogenerator for self-powered gesture recognition and language expression

**DOI:** 10.1080/14686996.2019.1665458

**Published:** 2019-09-11

**Authors:** Che-Min Chiu, Shuo-Wen Chen, Yu-Ping Pao, Ming-Zheng Huang, Shuen-Wen Chan, Zong-Hong Lin

**Affiliations:** a Institute of Biomedical Engineering, National Tsing Hua University, Hsinchu, Taiwan; b Department of Power Mechanical Engineering, National Tsing Hua University, Hsinchu, Taiwan; c Frontier Research Center on Fundamental and Applied Sciences of Matters, National Tsing Hua University, Hsinchu, Taiwan

**Keywords:** Gesture sensor, self-powered system, triboelectric nanogenerator, smart glove, language expression, 208 Sensors and actuators

## Abstract

Flexible electronics with great functional characteristics have proved to be a stepping stone in the field of wearable devices. Amongst all, gesture-sensing techniques have been widely studied for human-machine interfaces. In this paper, we propose a self-powered gesture-sensing system attached to the back of the hands, which has the capability of distinguishing hand gestures by measuring the triboelectric nanogenerator output signal. By attaching the sensor on the back of the hand, we can sense the displacement of tendons to detect the gestures. In addition, humidity resistance and durability of the device were tested and validated. Furthermore, we have established a set of rules to define the relationship between gestures and corresponding English letters. Therefore, the proposed sensor can further serve as an electronic sign language translator by converting gestures into words. Finally, we can integrate this system into gloves to enhance the applicability and utility. Overall, we have developed a real-time self-powered back-of-hand sensing system which can recognize various hand gestures.

## GRAPHICAL ABSTRACT

**Figure:**
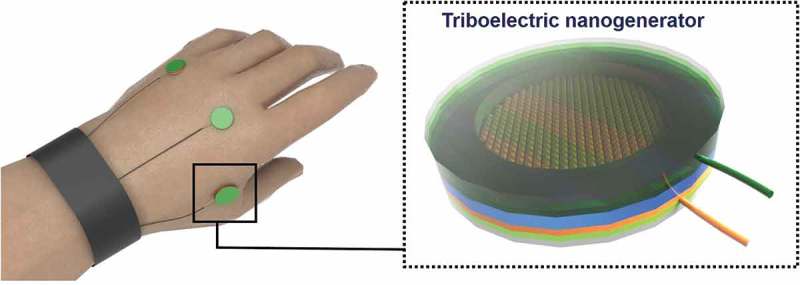

## 1. Introduction

In the past few years, wearable electronics for sensing human motions and gestures have drawn considerable attention due to their broad spectrum of applications in biomedical diagnostics, personal healthcare, human-machine interfaces, etc., [1–3]. Amongst various classifications, the field of sensors based on gestures is growing rapidly because of its vast potential to transform hand gestures into digitally processed controls for electronic devices. Currently, gestures are primarily recognized using the camera-based systems [4,5]. By utilizing embedded cameras to monitor fingertips and their locations, such systems achieve low latencies and high spatial resolution [3,6,7]. In addition, commercially available techniques like the electromyography (EMG) and inertial measurement units (IMUs) are also used for capturing hand movements [8]. However, these existing strategies can be expensive and make the sensor overweight if employed in the form of wearable devices. To improve the usability and practicality, a variety of nanomaterials with high conductivity have been used for the development of stretchable strain sensors in order to achieve wearable detection without using cameras [9,10]. However, one of the limitations for the majority of current wearable gesture sensors is that they rely on external power sources, which can cause inconvenience in terms of charging the device or even the lifetime of the battery [11]. In order to solve this problem, self-powered sensing systems came into play. Captivating strategies of self-powered systems have been recently investigated by the integration of energy harvesting devices in the form of nanogenerators. Amongst the various types, piezoelectric nanogenerators (PENG) have been extensively studied because they can transform mechanical energy from ambient vibrations into usable electrical energy, thus reducing the cost of operation [12–14]. However, the limited output performance remains a major challenge for such devices [15]. Compared to PENG, triboelectric nanogenerators (TENG) offer certain advantages like lightweight, smaller size, wider choice of materials for fabrication and better output performance, thereby making them promising candidates for integration with the flexible wearable electronics [16,17]. Nowadays, they have been widely developed as sustainable power sources for the detection of humidity [18], light [19], pressure [20], glucose [21], mercury ions [22], and so on. Meanwhile, the development of gesture sensing based on triboelectric effect has also made a major breakthrough. For example, Lee et al. reported a single-electrode mode TENG for recognizing finger movements [23]. They successfully designed a ﬂexible/wearable self-powered gesture sensor based on the charge transfer between the gold electrode and the epidermis, the outer layer of the skin. Despite the progress, there is still a room for improvement in the field of self-powered gesture sensors. Till date, the developed TENG-based gesture sensors are generally attached to the finger joints to monitor the random movements of the fingers [23–25], which in turn reduces dexterity of hands and increases discomfort [26]. In addition, the large bending angle may affect the durability of the sensors. In this paper, the drawbacks of the aforementioned examples have been addressed by placing the TENG-based gesture sensor on the back of hands. Since the flexor tendons of the fingers are connected to forearm muscles, the finger movements can also be observed on the back of hands. To validate our system, we placed three separate sensors on the tendons of the thumb, the index finger, and the little finger at the backside of the hand. By doing this, we were able to define eight different gestures from each hand by moving the three fingers and sticking them out to none, one, two, or all of them (three). Consecutively, there were 64 combinations of different gestures from two hands, which is sufficient for demonstrating 26 English alphabets for sign language recognition. Since the sensors were attached to the hands, various environmental factors like humidity would contribute significantly towards the sensor performance. As per our previous study, we have developed a chitosan-based TENG which has the capability of maintaining electric output even at a high relative humidity [26]. Therefore, in order to ensure the humidity independent performance and biocompatibility of the device, we chose chitosan/glycerol film as one of the triboelectric layers during fabrication [27]. In this work, for the first time, we have fabricated a self-powered gesture sensor which was fitted to the back of the hands to recognize the real-time finger motions. This research demonstrates its potential applications in sustainable wearable devices, self-powered electronic skin, and personalized medical devices.

## 2. Materials and methods

### 2.1. Preparation of the chitosan/glycerol film

For the preparation of the chitosan/glycerol film, different concentrations of glycerol were added to a solution containing 20 wt% chitosan in acetic acid. After addition, the solutions were vigorously stirred until a homogeneous mixture was obtained. Next, with the help of spin coating process, the chitosan/glycerol solutions were coated on a silicon v-groove substrate which contained nanostructured patterns. Then, the chitosan/glycerol coated silicon substrate was put into a vacuum chamber where the vacuum pump was used to remove residual bubbles from the coated substrate. Finally, the as-prepared sample was incubated in a hot circulator exact oven at 60°C for 4 h to form a chitosan/glycerol thin film of 100 μm thickness on the top of the nanostructured-contained silicon substrate. The formed chitosan/glycerol films were characterized by field-emission scanning electron microscopy (FESEM, JEOL JSM-7600F) to determine the surface morphology.

### 2.2. Stability test of the chitosan/glycerol film

For the stability test, chitosan/glycerol and fluorinated ethylene propylene (FEP) films were cut into small pieces (2 × 3 cm^2^) to fabricate TENG. In order to accurately control the operating frequency, a linear motor (LinMot H01) system was employed to make the TENG operate from 2 Hz to 10 Hz. By measuring the voltage output with a programmable electrometer (Keithley Model 6514), the durability of the device can be evaluated. In addition, to study the effect of humidity on the stability of TENG, the voltage output was measured in the presence of relative humidity of 20% to 80% using the programmable electrometer.

### 2.3. Gesture measurement and alphabet recognition

For gesture measurement and alphabet recognition, three TENG, each with a radius of 0.5 cm, were fabricated and attached to the back of the hand. The three sensors were placed on the tendons of each thumb, forefinger and little finger for each hand. When making a gesture, the electrical output of the three sensors was measured separately by a low-noise preamplifier (Stanford Research System Model SR560) and a low-noise current amplifier (Stanford Research System Model SR570).

## 3. Results and discussion

### 3.1. Fabrication of TENG

**Figure 1.**
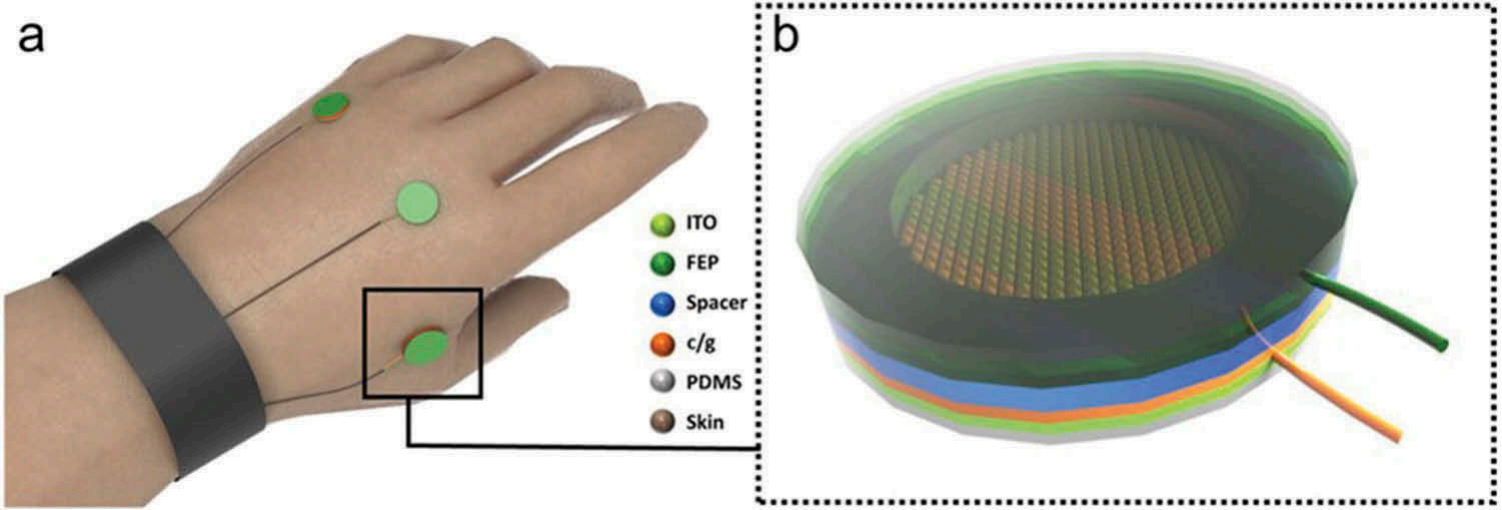
Structural design of the wearable self-powered gesture sensor. (a) Schematic illustration of the self-powered gesture sensor. (b) Enlarged image of the TENG.

As shown in Figure 1(b), the TENG was fabricated using ITO-FEP as one layer and chitosan/glycerol coated nanostructured substrate as the other layer separated from each other using a double-sided adhesive as the spacer layer. Briefly, an indium tin oxide (ITO, 130 nm)-coated polyethylene terephthalate (PET) substrate (25 μm thick) with highly transparent and flexible characteristics was attached to the 20 μm-thick polydimethylsiloxane (PDMS) substrates. Then, FEP film of 50 μm-thickness was enclosed on the top of the ITO sheet. Then, donut-shaped double-sided adhesive with a thickness of 50 μm was stuck between two triboelectric layers. In order to obtain the electrical output, two copper wires were connected to the ITO and chitosan/glycerol films. Finally, PDMS mixture with a weight ratio of 10 (base/curing agent) was dispensed onto the ITO films and cured at 60°C for more than 12 h to form the substrate for the device. Owning to its high biocompatibility, PDMS was chosen in this case to form the substrate for the TENG. A schematic of the production process is shown in Figure S1.

The as-formed biocompatible, flexible, transparent, and highly translucent TENG was then placed on the back of the hand, and each of them adheres closely to the skin for continuous monitoring (Figure 1(a)). It is worth noting that since the TENG is aimed to measure the deformation at the back of hands while the fingers move, therefore we placed the TENG sensors directly above the tendons.

### 3.2. Working principle

**Figure 2.**
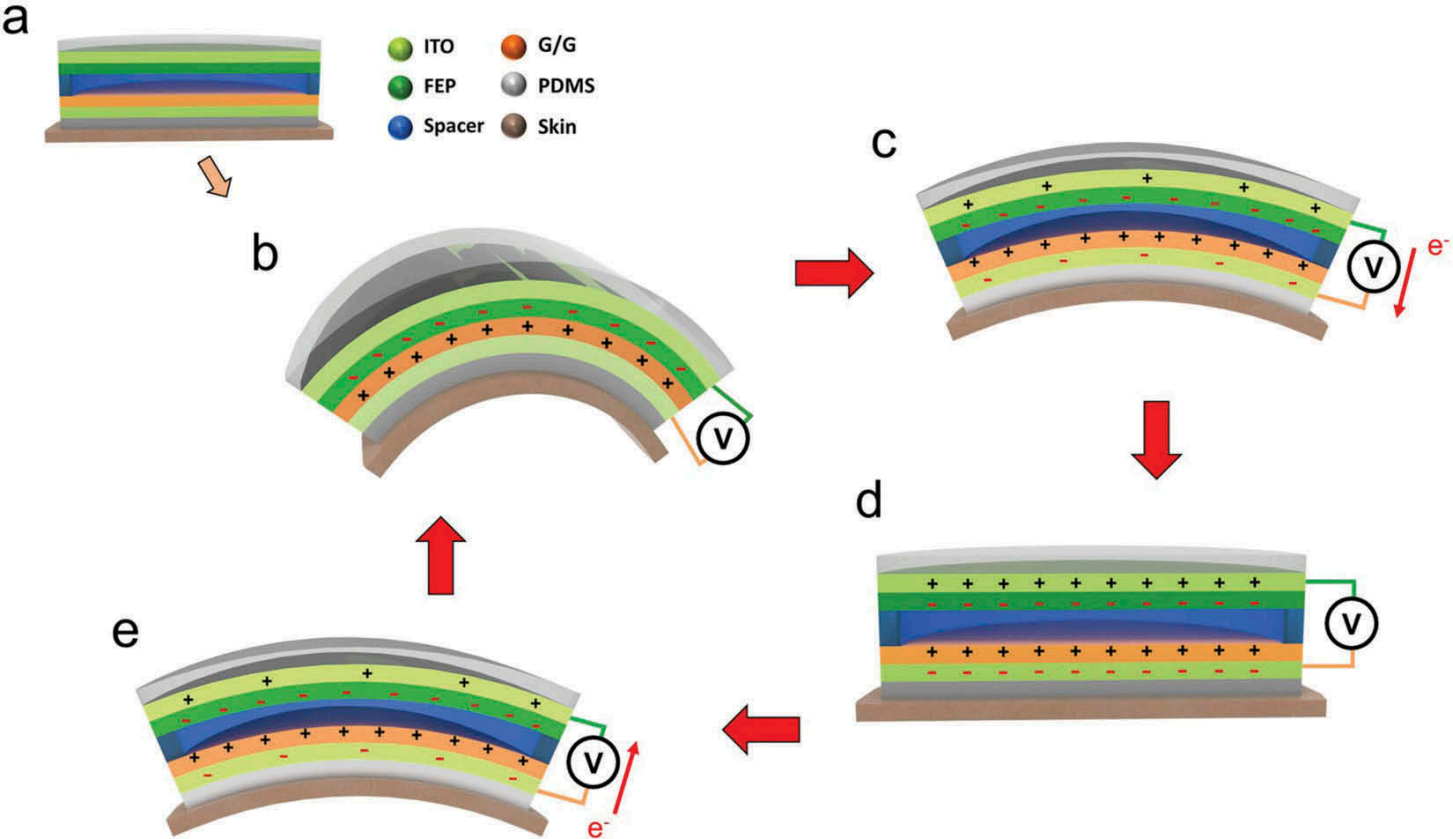
Working principle of triboelectric self-powered gesture sensor. (a-e) Schematic of the operating mechanism for gesture-sensing based on the triboelectric effect in a vertical contact-separation mode.

Although there is a high degree of correlation between the skin deformation at the back of the hand and the hand gesture, the physical changes occurring during the tendon’s oscillation are still negligible compared to the movement of the finger joints. Therefore, to accurately measure the surface deformation, the maximum oscillation amplitudes are highly required. We observed that when people first make a fist and then extend their fingers, the back of the hand could attain the maximum surface deformation. As a result, in the following experiments, we use the fist as the initial posture before making the gesture. As schematically illustrated in Figure 2, the three different fingers, namely thumb, the index finger, and the little finger, respectively, can lead to a significant displacement of the corresponding tendons when the fingers are extended. In order to harvest the mechanical energy provided by tendon movements, the TENG is designed to work in a contact-separation mode. A schematic diagram depicting the working principle is shown in Figure 2(a–e). During the first contact, the triboelectric effect will result in an equal amount of net negative charge on the FEP surface and net positive charge on the chitosan/glycerol surface as shown in Figure 2(b). When the fist is clenched, the skin on the back of the hand is relatively flat. Therefore, the two triboelectric layers of the device will be in a separated state (Figure 2(d)). Thereafter, the surface charges will create a potential difference between the two electrodes. Once a finger stretches out, the corresponding tendon will produce displacement and cause deformation on the skin surface. At this stage, both the interlayered distance and the potential difference will descend. As illustrated in Figure 2(e–b), in order to balance the potential difference, the electrons will flow from the ITO side to the chitosan/glycerol via the external circuit until the device is fully compressed. Finally, when the finger regains its original position, the gradual separation of the two layers will cause the increment of the potential difference which will generate a reverse electron flow in the external circuit until a new equilibrium state is obtained (Figure 2(c–d)). Therefore, through the periodic movement of the fingers, continuous outputs to determine different hand gestures can be obtained.

### 3.3. Electric output performance

**Figure 3.**
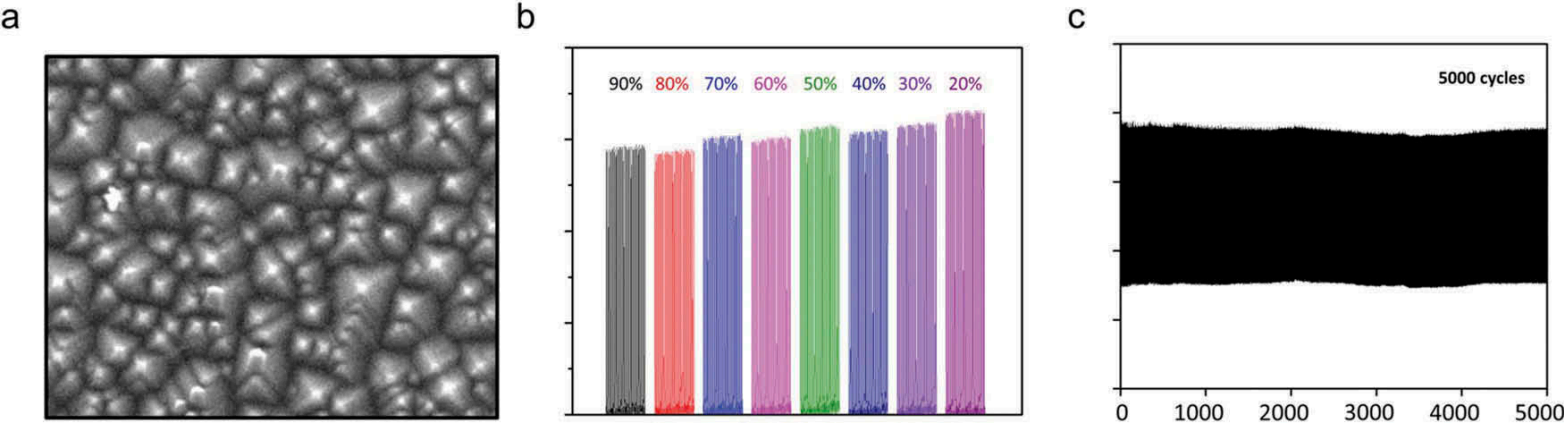
Characterization of the TENG. (a) SEM images of the chitosan/glycerol mixture. (b) A systematic of the open-circuit voltage generated from the chitosan-based TENG at different RH levels. (c) Durability test of the TENG under 5000 cycles.

According to the working principle of TENG, contact materials with rough surfaces are crucial for enhancing the electrical outputs. To promote the triboelectric effect, the chitosan/glycerol film is modified with nanostructures to increase the contact area. An SEM image of the chitosan/glycerol displayed in Figure 3(a) indicates the nano-pyramid-like arrays are present on the surface. Moreover, we compared the voltage output of the TENG with and without the presence of nanostructure (Figure S2). The results show that the voltage output of the TENG modified with the nanostructure is slightly larger than its unmodified counterpart. Subsequently, all the experiments were conducted using the nanostructure-modified TENG. In addition, humidity decreases the electrical output of TENG. Particularly, for wearable self-powered sensors that are attached to human skin, sweating can degrade the sensor performance. To solve this problem, we propose a chitosan-based TENG to generate a stable and high electric output under a wide range of humidity conditions. In our previous studies, it has been reported that the conductivity and relative permittivity of the as-prepared chitosan/glycerol film will increase with the rise in the relative humidity, which can offset the decrease in electrical output caused by moisture. During the measurement, the TENG is placed inside a humidity-controlled chamber. Figure 3(b) illustrates the effect of relative humidity ranging from 20% to 80% on the output voltage of the device. It can be clearly seen that the voltage remains constant irrespective of the increase in relative humidity (20% to 80%). The experimental result reveals that the applicability of the chitosan-based TENG in a wide range of environmental humidity conditions. Moreover, since the as-developed TENG will be utilized as a type of wearable sensor, it is important to investigate the flexibility of the electrode surface. In this regard, we have compared the effect of different resistances on the bending angle performance of the ITO electrode. As shown in Figure S3, the bending angle of the electrode remains unaffected by the different resistance values, thereby proving the conductive and flexible nature of the ITO electrode. Furthermore, we performed a durability test of the TENG to confirm the mechanical stability of the device. The output voltage (Figure 3(c) and Figure S4) of the TENG was measured at operating frequencies of 2, 5, and 10 Hz for 5000 cycles. The results show that there is no significant change in output voltage with the change in frequency which proves the robustness and mechanical durability of the device for practical applications.

### 3.4. Sign language system establishment

**Figure 4.**
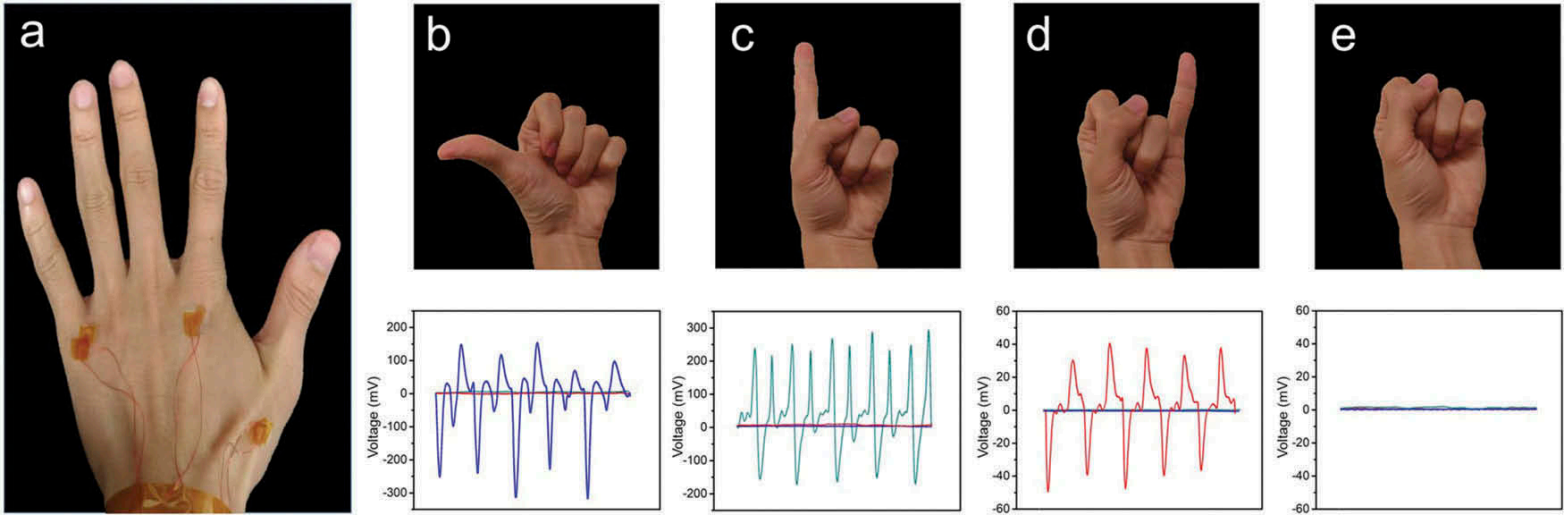
Electrical outputs of the TENG. (a) Photos of gesture sensors placed on the back of the hand. (b-e) Photographs of different gestures and their corresponding voltage outputs.

To demonstrate the capability of the device in sensing finger motions, three gesture sensors with a radius of 0.5 cm were attached to the back of the hand (Figure 4(a)). Since each of them is placed right above the tendons of the thumb, the index finger, and the little finger, respectively, only the movement of the corresponding finger can be detected. Moreover, the movements of the middle finger and the ring finger are noticeable while measuring other fingers. For this reason, sensors were not placed overhead the tendons of these two fingers. For the sensing experiment, two different start gestures states were compared: fully stretched state and the clenched fist state. In the former case, if the hand gesture is fully stretched initially, the movement of a single finger is more likely to interfere with the tendon corresponding to other fingers. On the other hand, if the initial gesture is in the state of a clenched fist, the movement of the finger is less likely to affect the tendon movement of other fingers. In order to elaborate this difference, we first measured the electrical output produced by the initial gesture of a clenched fist. As shown in Figure 4(b), when the fist gesture is maintained, none of the three sensors will detect the signal. Later, when we stretch out the thumb (Figure 4(c)), index finger (Figure 4(d)), and little finger (Figure 4(e)), respectively, the induced signal from different fingers can be detected independently. It can be clearly seen that the if a particular finger does not move, the corresponding sensor will not generate signal despite the movement of other fingers at the same time, demonstrating the high accuracy and sensitivity of the sensor. Additionally, different amplitudes of the output voltage can be attributed to the different deformations of the skin caused by different fingers. Moreover, it can be clearly observed that the deformation induced by the little finger is smaller than that of the other fingers, resulting in a relatively smaller output signal. Apart from this, as shown in Figure S5, the output current of the sensors were also measured. The result displays that the peak value of the generated current was around 4 nA when stretching out an index finger or thumb. But, the current signal generated by the little finger was only around 2 nA owing to the lesser displacement of the tendon. Following this, the electrical output produced by the initial stretched finger state was also evaluated (Figure S6). During the individual movement of the thumb, the signal generated can be easily recognized. However, when the index finger or the little finger was subjected to movement singly, the signal generated by them was interfered by the signal generated by other fingers. Also, the signals generated in the stretched state were also smaller than the group whose starting gesture was fist, which can be ascribed to the different displacements of the tendons under different initial gestures. Therefore, we decided to use the fist as the starting gesture for the subsequent experiments. In addition, to validate the stability of the device, we also measured the electrical output of the TENG after operating for 5,000 cycles at 2 Hz and 10 Hz. As shown in Figure S7, after 5,000 cycles, both of them displayed excellent gesture-sensing ability, which once again verifies that the stable nature of the device.

**Figure 5.**
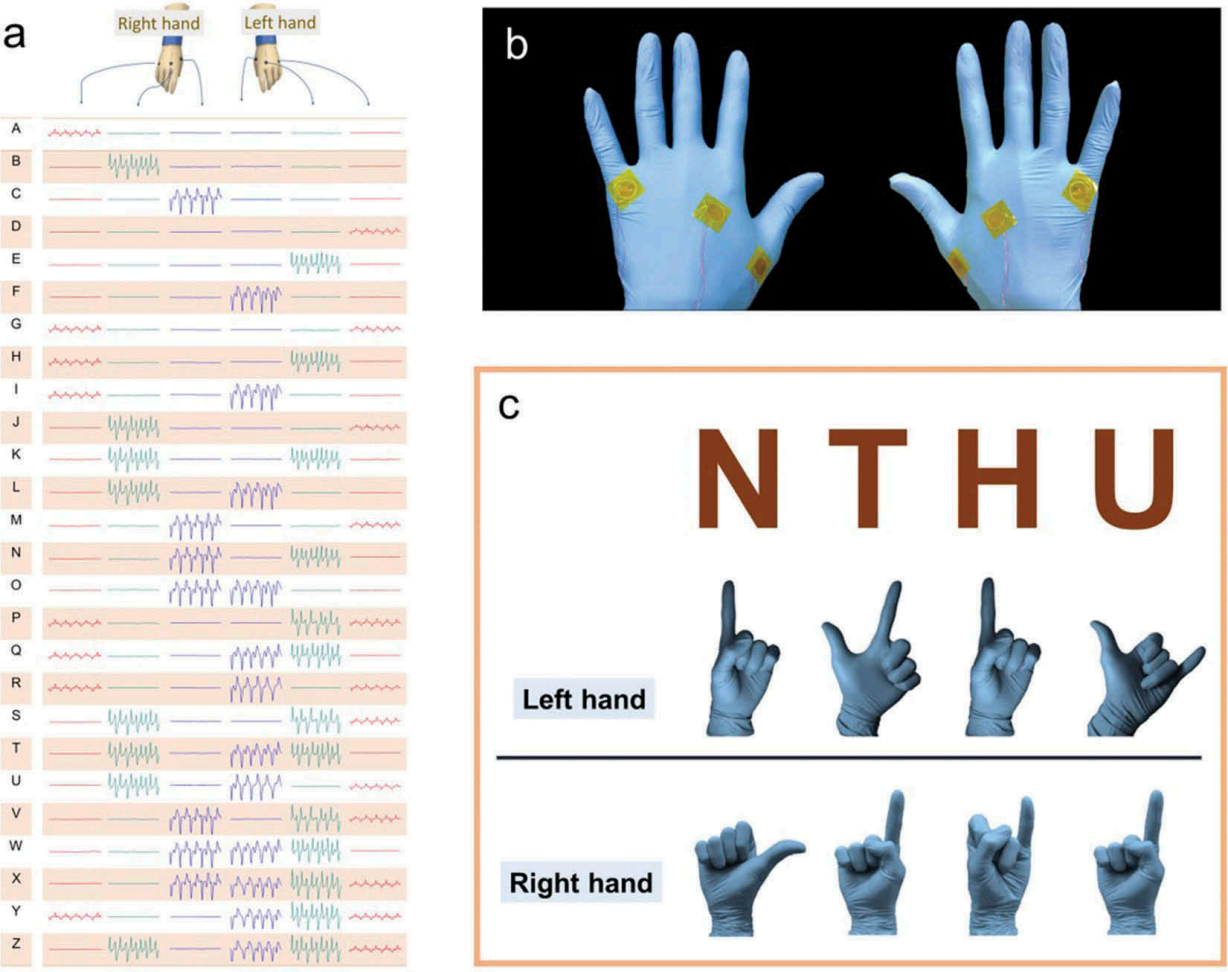
Practical application of sensors integrated into gloves. (a) English alphabet defined by gestures. (b) Photographic images of gesture sensors integrated into gloves. (c) Conversion of sign language into text.

As already mentioned, there were up to 64 combinations of gestures possible with both the hands together. As a result, we were able to demonstrate 26 different gestures as different English alphabets to create a new sign language system shown in Figure 5(a). For instance, the English letter H was defined by the left index finger and the right little finger. And, the English letter F was interpreted by the left thumb only. Hence, a self-powered sign language system was developed using the pattern on the back of the hand. Moreover, in order to demonstrate its practical application, we integrated the gesture sensors on the gloves, thus converting the regular gloves into smart ones (Figure 5(b)). It also indicates that the flexibility of the sensor allows for good conformity on the glove, thereby causing minimal sensitivity drop. In addition, Figure 5(c) displays an example of smart gloves by sensing different gestures to determine ‘NTHU’. The electrical signal induced from the smart gloves can be acquired when making aforementioned defined gestures. According to the proposed definition of alphabet N, only the left index finger and the right thumb remained in an extended state while other fingers remained in the bent state. Therefore, signal can only be generated from the extended fingers. Other letters also can be represented following the same rule. In this experiment, the identification of letters is still subjective, not programmed. Consequently, the developed self-powered gesture sensor demonstrates great potential in diverse applications, such as self-powered wearable medical devices, military communications systems, and other human-machine interface devices. This work provides important insights into designing smart keyboard combining interpretation programs with multi-channel systems to increase its practicability in the future.

## 4. Conclusions

In this paper, we have demonstrated the applicability self-powered gesture-sensing system fitted at the back of the hands. The chitosan-based TENG was rationally designed to obtain humidity independent triboelectric performance and to enhance the biocompatibility, which can, in turn, ensure stable signal and safety. By integrating the TENG onto the gloves which were capable of defining gestures, we have developed a low-cost system for converting gestures into sign languages. This system can effectively harvest the biomechanical energy from finger motions without affecting the comfort of the fingers. In addition, the device has proved to have excellent stability. Furthermore, our results disclose self-powered back-of-hand sensing techniques which can overcome the drawbacks of the reliance on existing external power-based or camera-based devices. Therefore, this ﬁnger-actuated wearable device could be a new way of recognizing hand gesture and be a prospective candidate to be used in human-machine interface due to numerous advantageous properties, such as lightweight, self-powered sensing, high flexibility, high comfort and scalability.
