# Supplementary material for: A smart glove with integrated triboelectric nanogenerator for self-powered gesture recognition and language expression
Source: Sci Technol Adv Mater. 2019 Sep 11;20(1):964–71. doi: 10.1080/14686996.2019.1665458 (PMC6781458; doi:10.1080/14686996.2019.1665458)
Supplement: Supplemental Material [file TSTA_A_1665458_SM5664.pdf]

A smart glove with integrated triboelectric nanogenerator for self-powered gesture recognition and language expression

Che-Min Chiu[1,#], Shuo-Wen Chen[1,#], Yu-Ping Pao[1], Ming-Zheng Huang[1], Shuen-Wen Chan[1] and Zong-Hong Lin[1,2,3,*]

[1] *Institute of Biomedical Engineering, National Tsing Hua University, Hsinchu 30013, Taiwan.*
[2] *Department of Power Mechanical Engineering, National Tsing Hua University, Hsinchu, 30013, Taiwan.*
[3] *Frontier Research Center on Fundamental and Applied Sciences of Matters, National Tsing Hua University, Hsinchu, 30013, Taiwan*

* Correspondence: linzh@mx.nthu.edu.tw; Tel.: +886-03-5715-131#35502
# These authors contributed equally to this work.

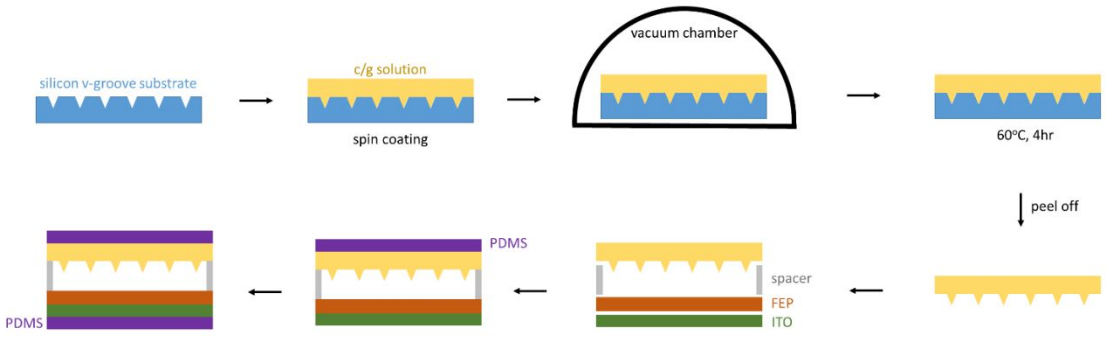

Figure S1. Fabrication process of the TENG. The chitosan/glycerol solutions were spin coated on a silicon v-groove substrate and then put into a vacuum chamber. Later, the sample was put into a hot circulator exact oven at 60 °C for 4 hours. Then, the c/g film was peeled off from the substrate and attached to a donut-shaped double-sided adhesive FEP film, and subsequently to an ITO film. Finally, PDMS mixture was dispensed onto the ITO films and cured at 60 °C to form the substrate for the device.

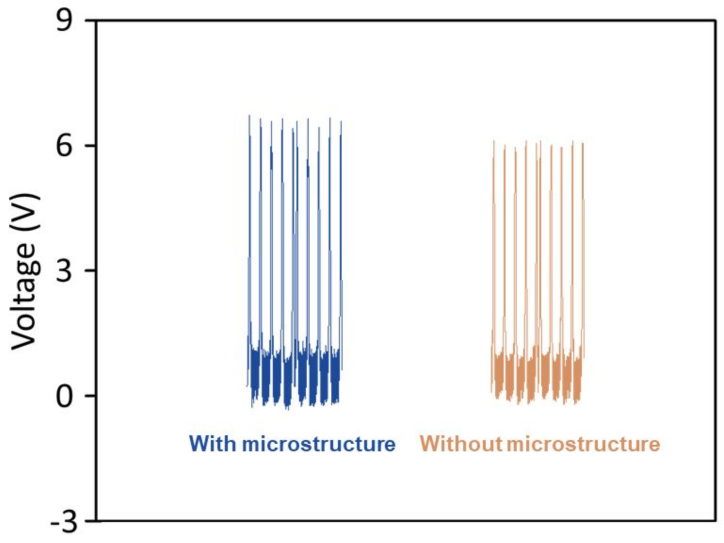

Figure S2. Measurement of electric output characteristics of the TENG with and without microstructure.

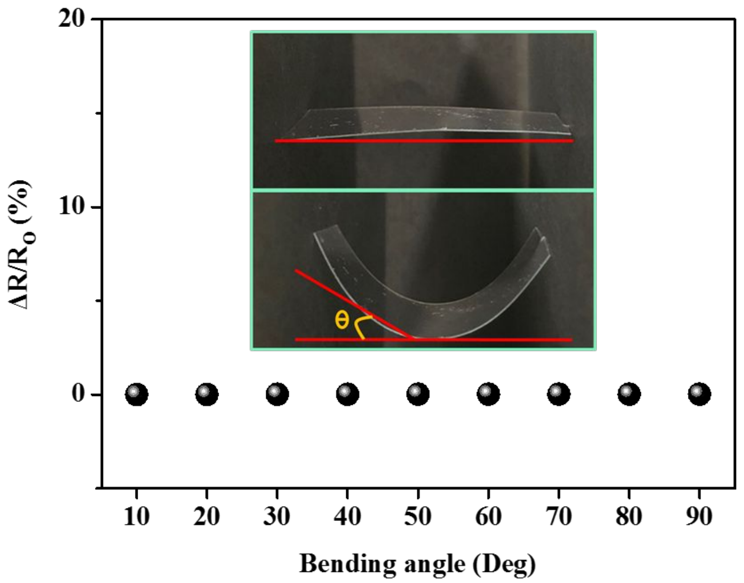

Figure S3. The effect of different resistance on the bending angle performance of the ITO electrode.

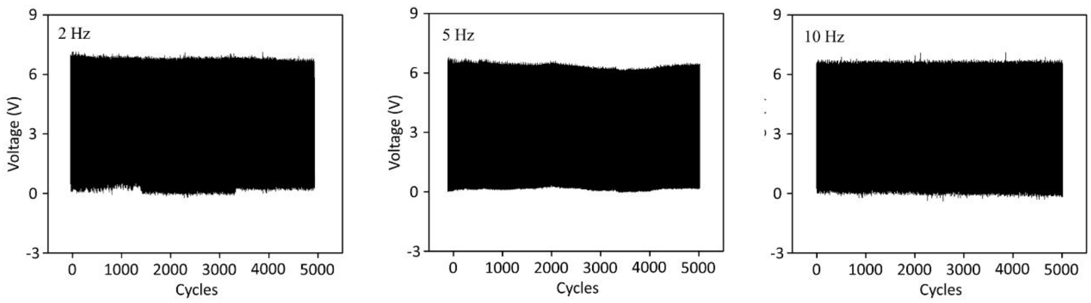

Figure S4. Durability test of the TENG under 5000 cycles when the operating frequency is at 2 Hz, 5 Hz and 10 Hz.

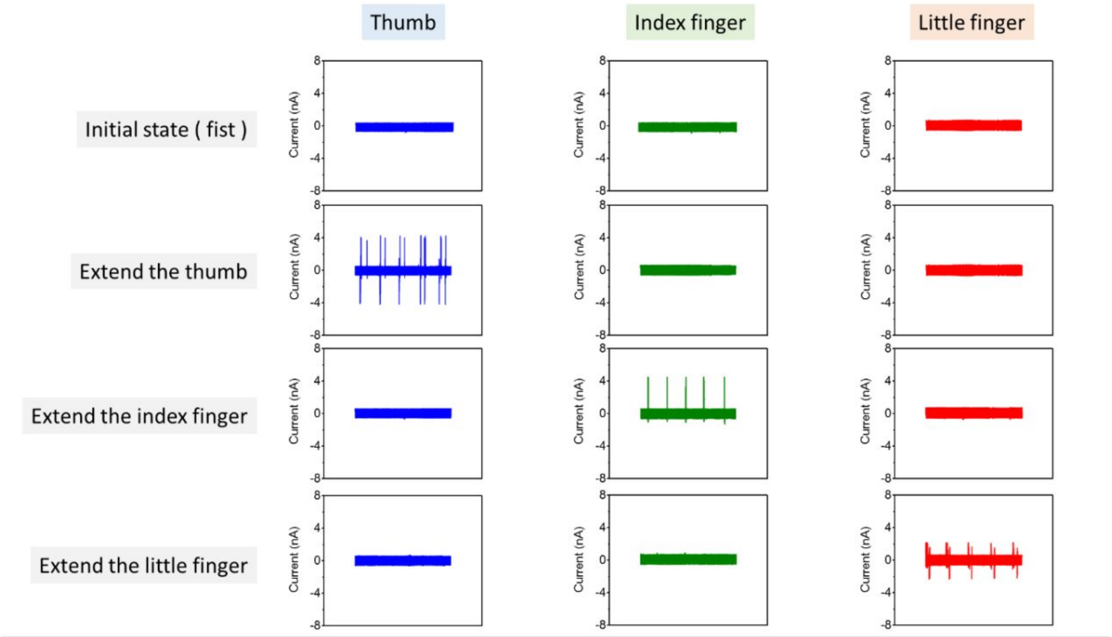

Figure S5. Outputs current of the three TENG mounted on the tendon corresponding to the thumb (blue), the index finger (green), and the little finger (red) when moving different fingers.

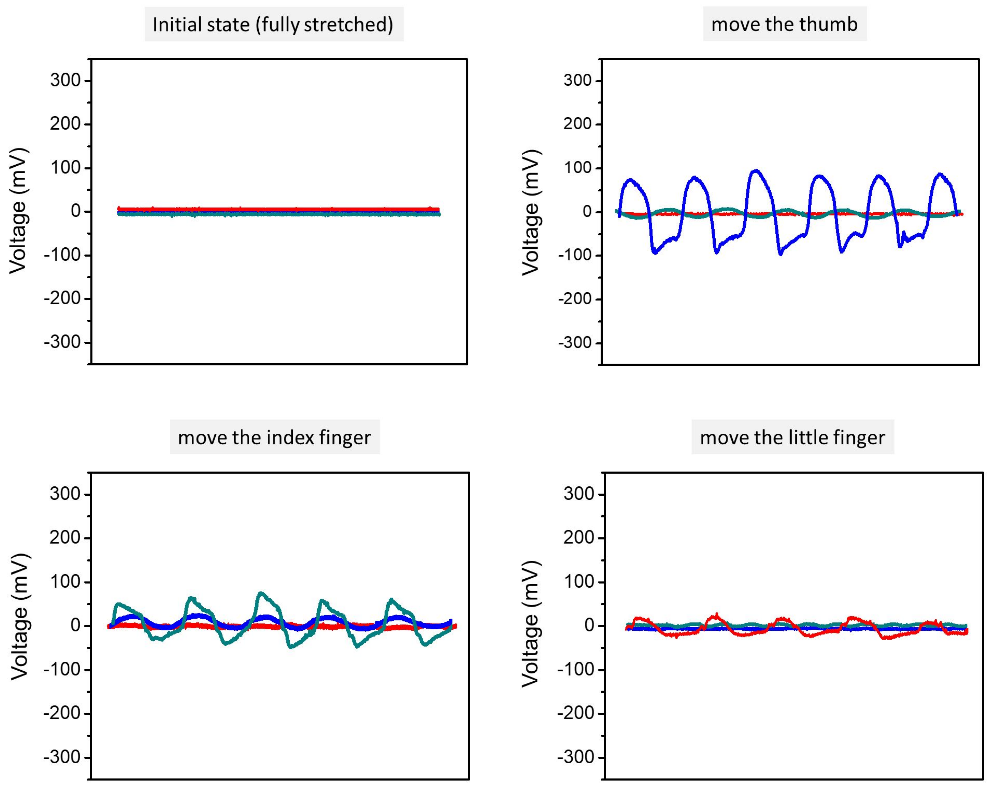

Figure S6. Electrical outputs of the three TENG mounted on the tendon corresponding to the thumb (blue), the index finger (green), and the little finger (red) when moving different fingers.

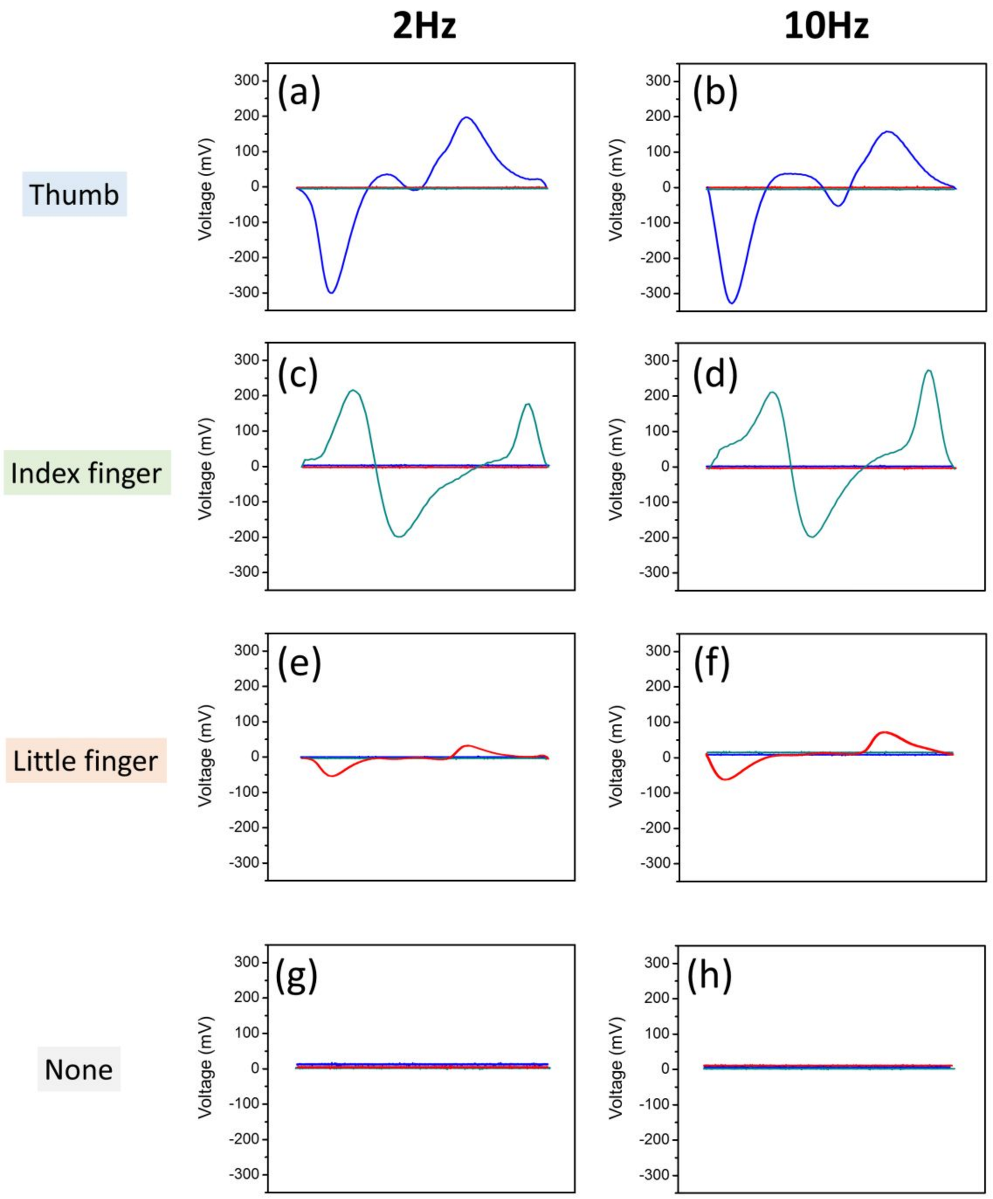

Figure S7. Electrical output of the TENG after operating 5000 cycles at 2 Hz and 10 Hz.
